# Broad spectrum of regorafenib activity on mutant *KIT* and absence of clonal selection in gastrointestinal stromal tumor (GIST): correlative analysis from the GRID trial

**DOI:** 10.1007/s10120-021-01274-6

**Published:** 2022-01-20

**Authors:** Michael Jeffers, Christian Kappeler, Iris Kuss, Georg Beckmann, Daniel H. Mehnert, Johannes Fredebohm, Michael Teufel

**Affiliations:** 1 Bayer HealthCare Pharmaceuticals, 100 Bayer Blvd, Whippany, NJ 07981 USA; 2 Bayer Pharma AG, Berlin, Germany; 3 Sysmex Inostics GmbH, Hamburg, Germany; 4 Present Address: Boehringer-Ingelheim Pharmaceuticals Inc., Ridgefield, CT USA

**Keywords:** Regorafenib, KIT, GIST, Mutation analysis

## Abstract

**Background:**

In the phase 3 GRID trial, regorafenib improved progression-free survival (PFS) independent of *KIT* mutations in exons 9 and 11. In this retrospective, exploratory analysis of the GRID trial, we investigated whether a more comprehensive *KIT* mutation analysis could identify mutations that impact treatment outcome with regorafenib and a regorafenib-induced mutation pattern.

**Methods:**

Archived tumor samples, collected at any time prior to enrollment in GRID, were analyzed by Sanger sequencing (*n* = 102) and next-generation sequencing (FoundationONE; *n* = 47). Plasma samples collected at baseline were analyzed by BEAMing (*n* = 163) and SafeSEQ (*n* = 96).

**Results:**

In archived tumor samples, 67% (68/102) had a *KIT* mutation; 61% (62/102) had primary *KIT* mutations (exons 9 and 11) and 12% (12/102) had secondary mutations (exons 13, 14, 17, and 18). At baseline, 81% of samples (78/96) had *KIT* mutations by SafeSEQ, including the M541L polymorphism (sole event in 6 patients). Coexisting mutations in other oncogenes were rare, as were mutations in *PDGFR*, *KRAS*, and *BRAF*. Regorafenib showed PFS benefit across all primary and secondary *KIT* mutational subgroups examined. Available patient-matched samples taken at baseline and end of treatment (*n* = 41; SafeSEQ), revealed heterogeneous *KIT* mutational changes with no specific mutation pattern emerging upon regorafenib treatment.

**Conclusion:**

These data support the results of the GRID trial, and suggest that patients may benefit from regorafenib in the presence of *KIT* mutations and without the selection of particular mutation patterns that confer resistance. The study was not powered to address biomarker-related questions, and the results are exploratory and hypothesis-generating.

**Supplementary Information:**

The online version contains supplementary material available at 10.1007/s10120-021-01274-6.

## Introduction

The majority of gastrointestinal stromal tumors (GISTs; 70–80%) have mutations in the *KIT* receptor tyrosine kinase gene resulting in constitutive ligand-independent activation of KIT intracellular signaling [1, 2]. *KIT* mutations are predominantly found in exon 11 (juxtamembrane domain), with some also found in exon 9 (extracellular domain). Of the remaining GISTs that lack a *KIT* mutation, a minority (5–10%) have activating mutations in platelet-derived growth factor receptor-α (*PDGFRA*), while 10–15% of GISTs have no detectable mutations in either *KIT* or *PDGFRA* (wild-type GISTs) [2]. Mutations in *KIT* and *PDGFRA* are usually mutually exclusive.

Tyrosine kinase inhibitors (TKIs) are the primary therapeutic option for the treatment of metastatic disease [1, 3]. The TKI imatinib demonstrated sustained activity in *KIT*-mutant GIST, thereby revolutionizing the treatment of GIST [2, 4]. However, many patients develop secondary resistance mutations in *KIT*, which can arise within the ATP-binding pocket (exons 13 and 14) or activation loop (exons 17 and 18) of the kinase domain [5–7]. Following progression on imatinib, patients can go on to receive treatment with the TKI sunitinib, which is active against some imatinib-resistant *KIT* mutations, but most patients eventually progress.

Regorafenib is an oral TKI that, together with its metabolites, demonstrates activity against a variety of kinases, including wild-type and mutant *KIT *in vitro and in vivo [8–12]. In the phase 3 GRID trial, regorafenib significantly improved the primary endpoint of progression-free survival (PFS; hazard ratio [HR] 0.27; 95% confidence interval [CI] 0.19–0.39; one-sided *P* < 0.0001) versus placebo in patients with advanced GIST who previously progressed on imatinib and sunitinib and who were unselected for mutation subtypes [13]. Based on these results, regorafenib was approved as third-line treatment of patients with advanced GIST after imatinib and sunitinib [14, 15], and regorafenib is included as a Category 1 preferred option for these patients in the NCCN Clinical Practice Guidelines in Oncology for GIST [1]. Regorafenib clinical benefit was demonstrated to be independent of detectable primary *KIT* mutations in exons 9 (HR 0.24; 95% CI 0.07–0.88) and 11 (HR 0.21; 95% CI 0.10–0.46) based on biomarker data at study entry from 66/199 patients in the GRID trial [13].

Using a liquid biopsy approach from specimens collected in the GRID trial, we performed a retrospective exploratory analysis to investigate whether *KIT* mutations (primary and/or secondary) might impact the treatment benefit of regorafenib, and whether a treatment-induced pattern of mutations could be identified.

## Materials and methods

### Study design

GRID (NCT01271712) was a randomized, double-blind, placebo-controlled phase 3 trial conducted at 57 centers in 17 countries. Efficacy and safety outcomes of the GRID trial have been reported elsewhere [13]. Briefly, patients with metastatic and/or unresectable GIST who had disease progression with at least prior imatinib and sunitinib were randomized (2:1) to receive oral regorafenib 160 mg once daily or matching placebo, each with best supportive care, for the first 3 weeks of each 4-week cycle. Tumor assessments were performed at baseline, then every 4 weeks for the first 3 months, every 6 weeks for the next 3 months, and every 8 weeks until end of treatment. Patients were treated until disease progression, unacceptable toxicity, or patient decision to withdraw from the trial. In the event of centrally assessed tumor progression, treatment assignment could be unblinded and patients receiving placebo offered to cross over to receive open-label regorafenib. The primary endpoint was PFS (modified Response Evaluation Criteria In Solid Tumors [RECIST] v1.1, by blinded central radiology review) and overall survival (OS) was a secondary endpoint. Biomarker evaluation was an exploratory endpoint.

### Biomarker sampling and assays

Archived biopsies and/or plasma samples were collected from patients who provided consent for biomarker analyses. Archived tumor samples were collected at any time prior to the start of GRID, while plasma samples were freshly collected at baseline (i.e., just prior to the start of treatment), and again at the end of treatment (EoT), in the GRID study.

#### Archived tumor tissue

DNA from archived formalin-fixed, paraffin-embedded tumor tissue specimens was analyzed by Sanger sequencing and next-generation sequencing (NGS). Bi-directional Sanger sequencing was performed on DNA isolated from tumor tissue specimens that were examined by a pathologist and contained at least 50% tumor cells. Tissue specimens judged to be composed of < 50% tumor cells were macro-dissected to enrich for tumor cell content prior to DNA isolation. Tissue specimens that were judged to contain < 50% tumor cells following macro-dissection were not included in the mutational analysis. Exons 9, 11, 13, 14, 17, and 18 in the *KIT* gene, exons 12, 14, 15, and 18 in the *PDGFRA* gene, exon 15 in the *BRAF* gene, and exon 1 in the *KRAS* gene were sequenced using the Sanger method as previously described [16]. NGS was performed on the FoundationOne panel of 280 tumor genes (Foundation Medicine, Inc.) as previously described [17].

#### Plasma samples

The amount of human genomic DNA isolated from plasma samples was quantified using a modified version of the human LINE-1 qRT-PCR protocol [18]. DNA is reported in genome equivalents, with one genome equivalent being one haploid human genome weighing 3.3 pg.

Circulating tumor-associated mutations in fresh plasma DNA (ctDNA), collected at baseline, were detected by BEAMing (Beads, Emulsions, Amplification, Magnetics) by Sysmex Inostics GmbH [19]. The BEAMing assays were designed to detect 29 tumor-associated mutations in the *KIT* gene (exons 9, 11, 17, and 18), 5 in *PDGFRA*, 1 in *BRAF*, and 7 in *KRAS*; the list of mutations analyzed is outlined in Table S1.

Tumor-associated mutations in exons 8–18 in the *KIT* gene from fresh plasma DNA, collected at baseline and at EoT, were also analyzed by Sysmex Inostics GmbH using a targeted next-generation DNA sequencing approach (SafeSEQ), allowing the detection of de novo missense mutations and insertions/deletions in the region covered by the amplicons. This approach followed the Safe Sequencing protocol as described previously [20], and provides the ability to detect mutant allele frequencies (MAF) down to 0.01% [21] depending on input amount and nucleotide position/change.

### Statistical analysis

The biomarker analysis in this study was exploratory and all findings are therefore hypothesis-generating rather than confirmatory. PFS and OS were estimated using the Kaplan–Meier method, with HR and 95% CI calculated using a Cox regression model. No adjustment for multiplicity was performed. Average tumor burden between cfDNA quartiles was compared using a *t* test.

## Results

### Patient disposition and demographics

**Fig. 1:**
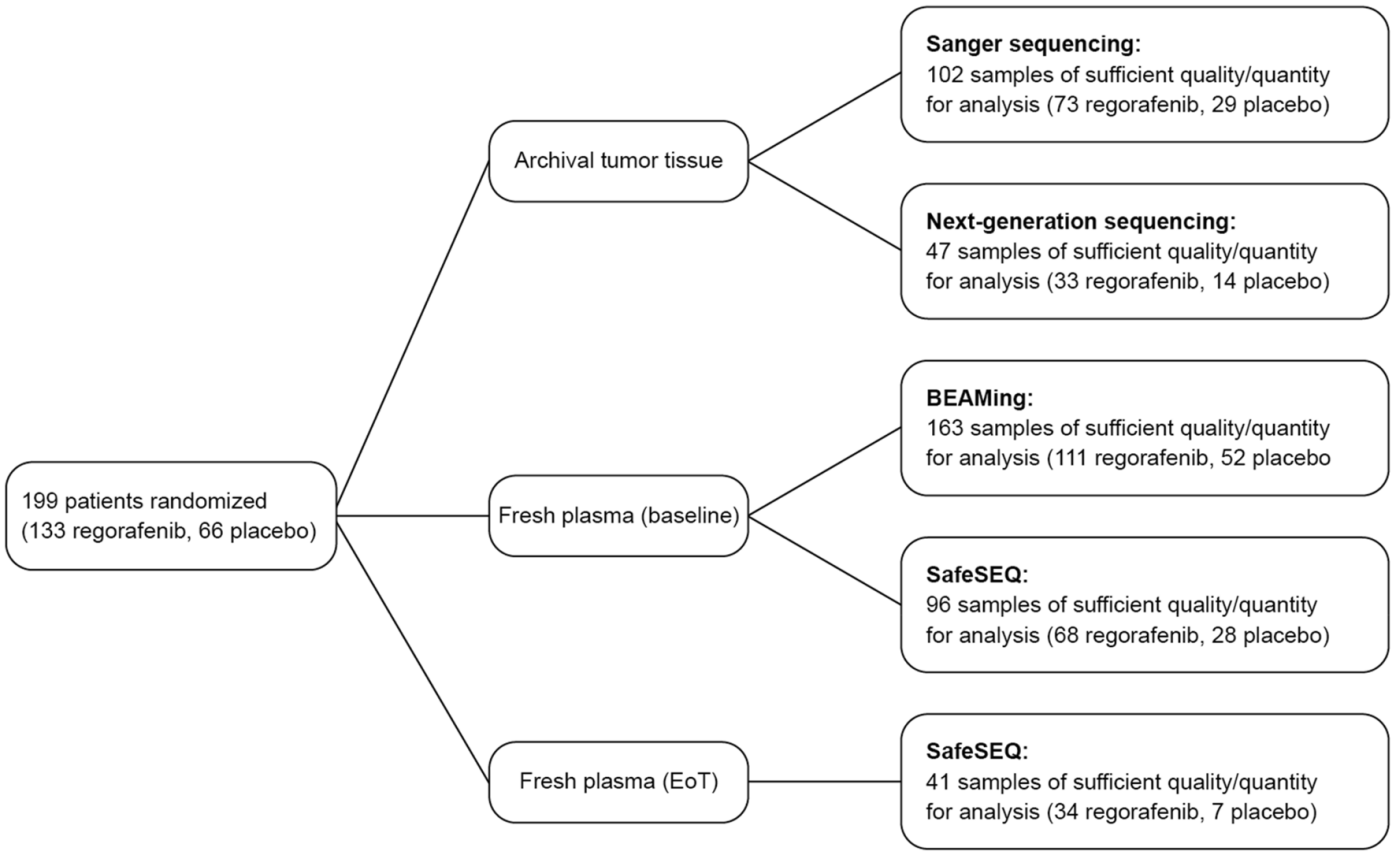
GRID patient subgroups for biomarker analyses. *EoT* end of treatment

In the GRID trial, 199 patients were randomized to receive regorafenib (*n* = 133) or placebo (*n* = 66) between January and August, 2011; one patient in the regorafenib group did not receive treatment [13]. Tissue and plasma samples for biomarker analysis of appropriate quality were available for a subset of patients (Fig. 1). The tissue specimens were from samples that had been collected at various time points prior to study entry, whereas the plasma samples were freshly collected at baseline (i.e., just prior to the start of treatment).

Patient demographics and disease characteristics at baseline in the overall GRID population and BEAMing biomarker cohort were generally similar, while more variation was observed in the SafeSEQ cohort due to the small sample size (Table 1). The PFS and OS treatment benefit in the biomarker subgroups were similar to the overall cohort, apart from slight variation in OS values for the smaller SafeSEQ cohort (Table 2).

**Table 1:** Patient demographics and baseline characteristics for the overall GRID population and biomarker cohorts

Variable	Overall GRID cohort (*N* = 199)		BEAMing^a^ (*n* = 163)		SafeSEQ^a^ (*n* = 96)	
	Regorafenib (*n* = 133)	Placebo (*n* = 66)	Regorafenib (*n* = 111)	Placebo (*n* = 52)	Regorafenib (*n* = 68)	Placebo (*n* = 28)
Median age, years (range)	60 (18–82)	61 (25–87)	60 (18–82)	61 (25–87)	57 (18–80)	60.5 (30–84)
Sex, *n* (%)						
Male	85 (64)	42 (64)	72 (65)	31 (60)	48 (71)	16 (57)
Female	48 (36)	24 (36)	39 (35)	21 (40)	20 (29)	12 (43)
Race, *n* (%)						
White	90 (68)	45 (68)	83 (75)	37 (71)	51 (75)	20 (71)
Asian	34 (26)	16 (24)	19 (17)	10 (19)	9 (13)	5 (18)
Black or AA	0	1 (2)	0	1 (2)	0	1 (4)
NR or missing	9 (7)	4 (6)	9 (8)	4 (8)	8 (12)	2 (7)
ECOG PS, *n* (%)						
0	73 (55)	37 (56)	60 (54)	29 (56)	39 (57)	16 (57)
1	60 (45)	29 (44)	51 (46)	23 (44)	29 (43)	12 (43)
Previous systemic anticancer therapy, *n* (%)						
2 lines	74 (56)	39 (59)	59 (53)	31 (60)	31 (46)	19 (68)
> 2 lines	59 (44)	27 (41)	52 (47)	21 (40)	37 (54)	9 (32)
Duration of prior imatinib therapy (months), *n* (%)						
< 6	18 (14)	4 (6)	15 (14)	3 (6)	9 (13)	2 (7)
6–18	26 (20)	7 (11)	23 (21)	6 (12)	12 (18)	3 (11)
> 18	89 (67)	55 (83)	73 (66)	43 (83)	47 (69)	23 (82)

*AA* African American; *ECOG* *PS* Eastern Cooperative Oncology Group performance status; *NR* not reported

^a^BEAMing and SafeSEQ data are from baseline plasma samples; SafeSEQ data from end of treatment plasma samples are not included due to the small sample size (*n* = 41)

**Table 2:** Treatment effect in the overall GRID patient population versus biomarker cohorts

Population	*N*	Progression-free survival HR (95% CI)	Overall survival HR (95% CI)
Overall population [13]	199	0.27 (0.19–0.39)	0.77 (0.42–1.41)
BEAMing biomarker cohort	163	0.29 (0.19–0.43)	0.79 (0.41–1.51)
SafeSEQ biomarker cohort	96	0.27 (0.16–0.47)	0.90 (0.38–2.09)

*CI* confidence interval; *HR* hazard ratio

### Oncogenic mutations in GIST

We analyzed the mutational status in archival tumor tissue and fresh baseline plasma using different mutation-detection methods (Table 3). Initially, archival tumor samples were analyzed by Sanger sequencing for mutations in *KIT*, *PDGFRA*, *BRAF*, and *KRAS*. A *KIT* mutation was identified in 67% (68/102) of tumor samples acceptable for analysis (≥ 50% tumor cells); 61% (62/102) had primary mutations, 12% (12/102) had secondary mutations, and 33% (34/102) were *KIT* wild type. Mutations in the other analyzed oncogenes were rare: *PDGFRA* mutations were found in 3 samples (3%), activating *KRAS* mutations were found in 2 samples (2%), one of which did not contain a *KIT* mutation, and none of the samples had a *BRAF* mutation. No mutations in *KIT*, *PDGFRA*, *KRAS*, or *BRAF* were identified in 28% of samples (29/102). The different *KIT* mutational status in archived biopsies versus plasma samples taken at baseline (Table 3) may partly reflect the differences in prior treatment, with archival biopsies taken prior to treatment with regorafenib and other TKIs (before imatinib/sunitinib treatment) resulting in a lower frequency of secondary *KIT* mutations.

**Table 3:** Summary of *KIT* genotyping data^a^

*KIT* alteration	Archival tumor tissue (at any time prior to start of regorafenib in GRID)	Plasma samples (at baseline, just prior to start of regorafenib in GRID)	
	Tissue Sanger sequencing *N* = 102	Plasma BEAMing *N* = 163	Plasma SafeSEQ *N* = 96
Any, *n*/*N* (%)	68/102 (67)^‡^	94/163 (58)^‡^	78/96 (81)^†^
Primary, *n*/*N* (%)	62/102 (61)	43/163 (26)	Exon 8: 3/96 (3%)^¶^ Exon 9: 14/96 (15%) Exon 11: 26/96 (27%)
Secondary, *n*/*N* (%)	12/102 (12)	77/163 (47)	Exon 13: 15/96 (16%) Exon 14: 7/96 (8%) Exon 17: 43/96 (45%) Exon 18: 13/96 (14%)

^a^The BEAMing assays utilized in this study provided incomplete coverage of the numerous primary *KIT* alterations that have been reported in GIST, but good coverage of reported secondary *KIT* alterations

^‡^The M541L variant in exon 10 is not included

†The M541L variant in exon 10 is included

^¶^It is unknown whether exon 8 mutation is a primary mutation and of clinical significance

The archival tumor samples were also analyzed by NGS for the presence of mutations in a number of oncogenes, including *KIT*. Targeted tumor NGS was performed on 47/102 available samples using the FoundationOne assay on a panel of 280 known oncogenes. A *KIT* mutation was detected in 85% of samples (40/47) and secondary mutations (in exons 13/14 or 17/18) were present in 26% of samples (12/47). The higher overall mutation rate with NGS, versus Sanger sequencing, may be explained by the higher analytical sensitivity of the NGS method [22]. To confirm this, 30/47 samples were analyzed by both Sanger sequencing and FoundationOne. Discordance between the data sets was found in 17% of samples (5/30), in three of which an additional mutation was detected by NGS. In the 47 samples analyzed by NGS, coexisting oncogenic somatic mutations were rare and identified in genes such as *PI3K*, *MLL2*, *NF1*, *NF2*, *TP53*, *HRAS*, and *ErbB4* (1 each; Fig. S1); mutations in *PDGFRA* were absent from this sample set. Other noteworthy oncogenic driver deletions were identified in *CDKN2A* (18/47) and *RB1* (6/47). Three *KIT* wild-type samples had mutations in other oncogenes (Fig. S1), but in 4 patients no oncogenic mutations were detectable.

In summary, these results highlight the benefit of using ctDNA liquid biopsies versus archival tumor tissue, and for using sensitive analytical methods.

### Baseline circulating DNA levels and association with tumor burden and outcome

To assess if baseline circulating-free plasma DNA (cfDNA) levels could predict tumor burden and outcome, plasma cfDNA levels from 162 patients were analyzed for correlation with tumor burden. Using a quartile analysis, a potential association was identified between high cfDNA levels and increased tumor burden (average tumor burden in the last quartile to that in the first quartile, *P* = 0.042; Table S2). However, no correlation between baseline cfDNA levels and treatment benefit (PFS or OS) with regorafenib was observed (Table S2).

### *KIT* mutational analysis of baseline circulating DNA

The *KIT* mutational spectrum in ctDNA was assessed by BEAMing in plasma collected at baseline from 163 of 199 patients (82%). *KIT* alterations were detected in 58% of patients (94/163); 26% (43/163) were in exons 9 and 11 and were considered to be primary driver mutations, while 47% (77/163) were presumptive secondary resistance mutations (exons 13, 14, 17, and 18) (Table 3). Most of the secondary mutations (64%, 49/77) occurred in exons 17/18 (activation loop), which are associated with exposure and resistance to sunitinib or imatinib [23]. All secondary mutations in patients with a concurrent *KIT* exon 9 alteration (*n* = 12) were located in the activation loop. Of note, 40% of patients (31/77) in whom a secondary mutation was identified had multiple secondary mutations, which could either be due to the presence of multiple mutations per lesion or a representation of tumor heterogeneity [24]. Similar to the archival tumor samples, mutations in *PDGFRA* or *BRAF* were rare (*n* = 2 and *n* = 0, respectively). A *KRAS* mutation was identified in one of the two patients in whom a *KRAS* mutation was identified in tumor tissue.

Since the *KIT* BEAMing assay was designed to detect a predetermined set of mutations, the remaining plasma samples with sufficient material (96 of 163 samples tested by BEAMing) were used to expand coverage of *KIT* mutations using the targeted NGS SafeSEQ technique. SafeSEQ detects all mutational hotspots and regions harboring presumptive secondary resistance mutations, while also allowing de novo detection of mutations in the respective genes. Using SafeSEQ, a *KIT* mutation was detected in 81% of samples (78/96) and multiple *KIT* mutations (up to 13) were found in most samples. Primary mutations were identified in exons 9 (*n* = 14) and 11 (*n* = 26), and secondary mutations in exons 13 (*n* = 15), 14 (*n* = 7), 17 (*n* = 43), and 18 (*n* = 13) (Table 3). As shown in Table S3, an NGS-based approach is particularly useful in regions that are of heterogeneous mutational background, such as deletions in exon 11, or in the presence of a large variety of missense mutations in the same codon (e.g., D820 in exon 17). In several cases, not all mutations detected by BEAMing could be verified by SafeSEQ, which may be due to different levels of input DNA. Presumptive primary mutations in *KIT* were also detected with SafeSEQ in exon 8 (*n* = 3), which was not included in the BEAMing analysis. Interestingly, a *KIT* M541L polymorphism in exon 10 with yet unknown oncogenic function was detected in 24% of patients (23/96), the majority of whom were heterozygous (20/25). In six samples, the M541L variant was the only alteration found. M541L is the most common *KIT* polymorphism known (rs3822214) with a minor allele frequency of 0.08 in the gnomAD database (https://gnomad.broadinstitute.org/), is classified as benign/likely benign on ClinVar (www.ncbi.nlm.nih.gov/clinvar/), and has been described in the literature as a driver of pediatric mastocytosis [25]. In patients with GIST, the *KIT* M541L polymorphism has been shown to be associated with a higher risk of metastasis at diagnosis, and with a higher risk of relapse [26, 27]. A higher prevalence of this genotype has also been seen in the disease of higher-risk patients (with tumor duality, un-resectable and/or locally advanced disease, in addition to metastases at diagnosis) compared with patients with localized GIST [27]. In that study, a positive correlation between *KIT* M541L occurrence and earlier onset of relapse in *PDGFRA* and wild-type GIST subgroups was found [27].

### Correlations between *KIT* mutational status and patient outcome with regorafenib

**Fig. 2:**
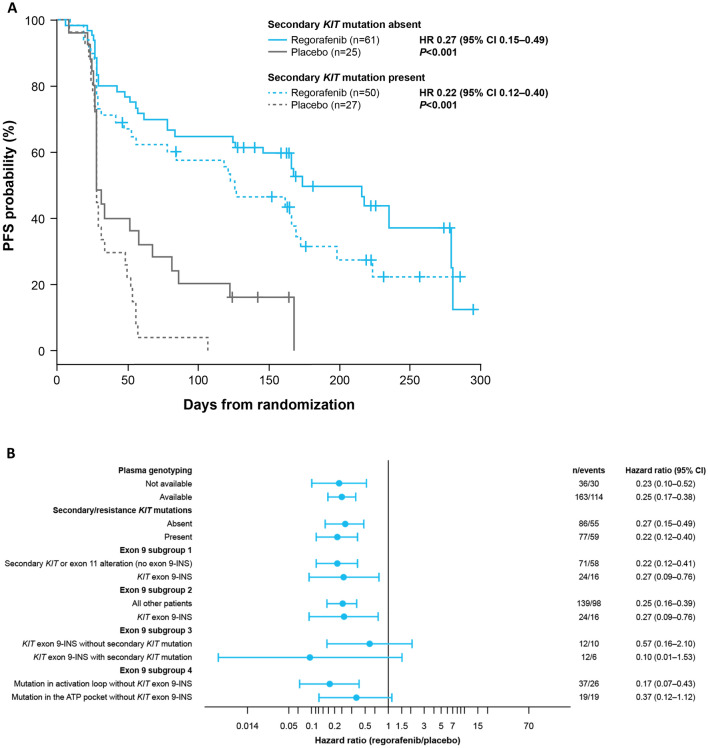
PFS of regorafenib vs placebo according to **A** the presence or absence of secondary *KIT* mutations and **B** *KIT* mutation subgroups as determined by BEAMing of plasma DNA (PFS from central assessment). *CI* confidence interval; *HR* hazard ratio; *INS* insertion; *PFS* progression-free survival

Potential association between *KIT* mutational status and clinical response (PFS) to regorafenib was evaluated. Treatment with regorafenib resulted in longer PFS versus placebo, regardless of whether a secondary mutation was present (HR 0.22, 95% CI 0.12–0.40; *P* < 0.001) or absent (HR 0.27, 95% CI 0.15–0.49; *P* < 0.001) in plasma DNA as assessed by BEAMing (Fig. 2A). Regorafenib showed PFS benefit within all mutation subgroups examined across both primary and secondary *KIT* mutations (Fig. 2B). In addition, regorafenib was associated with PFS benefit versus placebo in the presence (HR 0.046, 95% CI 0.004–0.605) and absence (HR 0.39, 95% CI 0.189–0.805) of the M541L polymorphism in exon 10 of the *KIT* gene, as determined by SafeSEQ (Fig. S2). Albeit based on a very small data set, our data showed that patients carrying the M541L variant had poor prognosis in the absence of treatment with regorafenib, and had shorter time on treatment with prior imatinib (median 878.5 days) and sunitinib (median 155 days) than those carrying M541 (median 925 and 230.5 days, respectively). This polymorphism was the only alteration found in exon 10.

### Longitudinal *KIT* mutational analysis

**Fig. 3:**
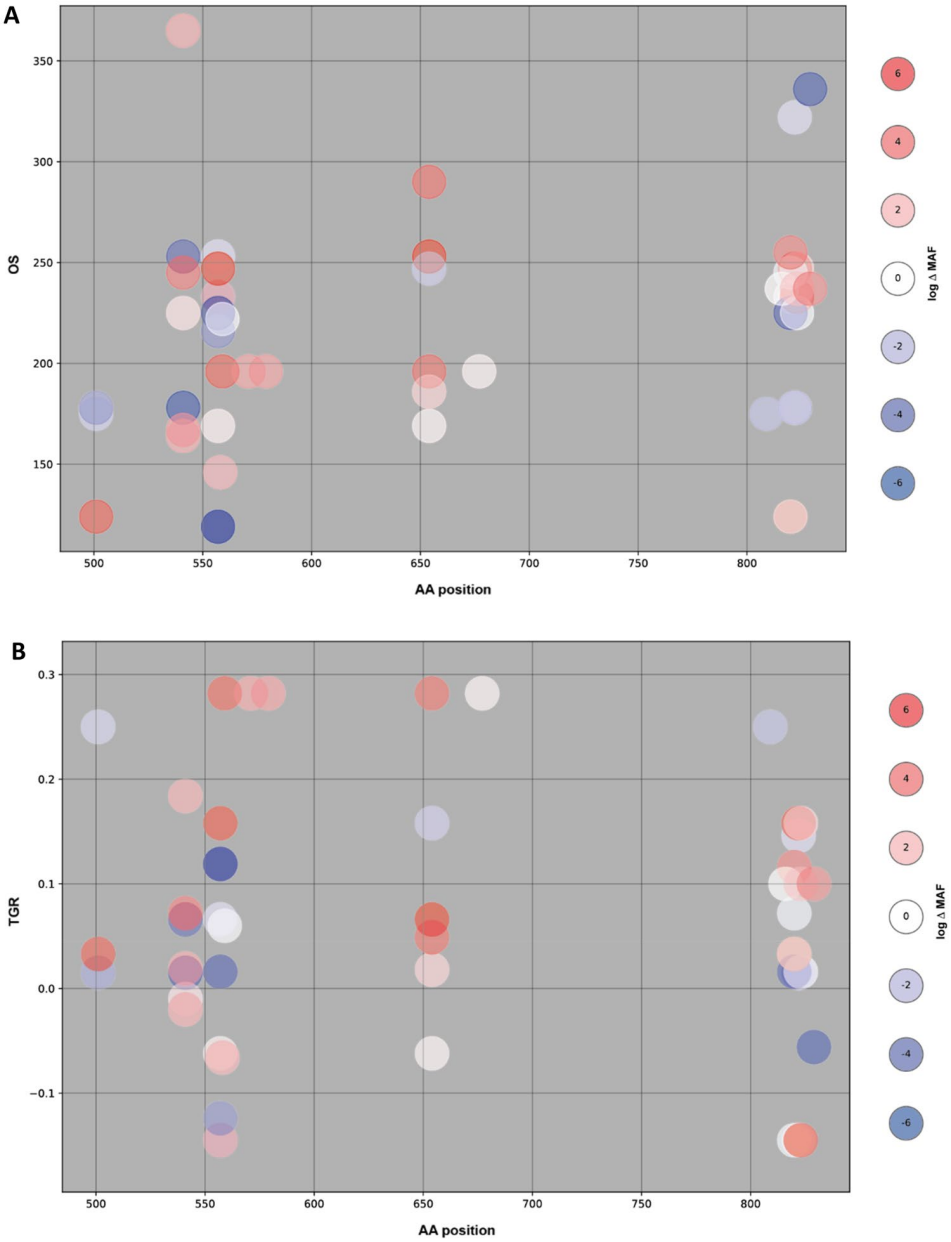
Treatment-induced changes in *KIT*-MAF and their relationship with OS (**A**) and TGR (**B**). Log differences in MAF induced during treatment with regorafenib are plotted on AA position (x-axis) and OS or TGR (y-axis). Negative changes (blue) represent decreasing MAF and positive changes (red) increasing MAF over treatment time. *AA* amino acid; *MAF* mutant allele fraction; *OS* overall survival; *TGR* tumor growth rate

To evaluate whether treatment with regorafenib results in induction of potential resistance mutations, which is best described by an increase in mutant allele fraction at EoT versus baseline, available individual patient baseline and EoT plasma samples (*n* = 41) were examined for *KIT* mutations by SafeSEQ. Duration of regorafenib treatment for patients with matched baseline and EoT samples ranged from 55 to 1131 days (1.8–37.2 months). Although in many cases, there was an increase in the mutant allele fraction across the *KIT* mutations at EoT versus baseline, the results did not show a trend for an increase of particular mutations that could act as a surrogate for resistance to regorafenib. The mutation analysis revealed a variety of changes in the *KIT* tumor genotype during treatment that were heterogeneous with no specific mutation pattern or association with outcome (OS) or tumor growth rate (Fig. 3). Some of the changes during treatment include (Table S4): the appearance of an exon 17 secondary mutation (Y823D) in a patient undergoing regorafenib treatment for > 3 years (patient 1); conversion from wild type to exon 17 mutation (D820Y/G) following long-term regorafenib treatment (679 days [22.3 months]; patient 10); disappearance of a primary mutation (exon 11, 557delWK) and strong reduction of secondary mutation allele frequency (D820G) following long-term regorafenib treatment (442 days [14.5 months]; patient 14); strong enrichment of exon 13 mutation (V654A) following long-term regorafenib treatment (433 days [14.2 months]; patient 15); and enrichment of a rare secondary mutation (D820H) following short-term regorafenib treatment (55 days [1.8 months]; patient 27). Interestingly, there was no consistent trend in the conversion of wild type to mutant *KIT* during treatment, with 7 out of the 11 *KIT* wild-type patients at baseline remaining wild type at the conclusion of treatment with regorafenib. One of these patients who was treated for 124 days had a *PDGFR* mutation (A842V). It is noteworthy that, unlike what has been described for imatinib or sunitinib [2, 5, 23, 28], the appearance of secondary mutations was rarely observed (e.g., D820 missense mutation in patient 10). Although intra-patient heterogeneity of biopsy sample-derived mutational profiles cannot be ruled out, these data suggest that progression during regorafenib treatment may be due to other yet-to-be-identified reasons, but unlikely by induction of clones harboring particular *KIT* exon 17 mutations.

## Discussion

The results presented here confirm the now well-established observation that the majority (up to around 80%) of GISTs harbor *KIT* mutations, and the rapid translation of these mutational data into effective targeted kinase inhibitor therapies has borne out their importance in GIST pathogenesis [2, 29]. Although being the second-most frequently mutated oncogene in GIST (up to 10%), mutations in *PDGFR* were rare in this study, which could be due to the fact that *PDGFRA-* mutated GISTs tend to have a lower risk of recurrence [30]. Preclinical results have shown that regorafenib is active against a number of primary or secondary *KIT* mutations, and is able to overcome treatment failure after imatinib and sunitinib [8, 10, 11]. The clinical benefit with regorafenib regardless of *KIT* status observed in the GRID trial [13] supported this hypothesis, and we therefore conducted this retrospective biomarker study to confirm this observation on a molecular level.

Using two analytically highly sensitive liquid biopsy ctDNA assays (BEAMing and SafeSEQ), both of which reliably allow the for the determination of true *KIT* mutational status in patients with GIST in the third-line setting, the presence of multi-clonal lesions harboring *KIT* mutations after prior TKI treatment was seen. The analysis did not show any differential efficacy effects for regorafenib on the examined mutational subgroups (primary exon 9 or exon 11 mutations, secondary exon 13/14 or exon 17/18 mutations, or combinations thereof). The activity of regorafenib in the presence of secondary exon 17 *KIT* mutations has additionally been demonstrated in a small clinical trial [31]. It should be noted that secondary *KIT* mutations in the activation loop (exons 17/18) were much more prevalent in this study than those in the ATP-binding pocket (exons 13/14). In fact, BEAMing of fresh baseline ctDNA did not identify a single case harboring a secondary mutation in the ATP-binding pocket in the context of a primary *KIT* exon 9 mutation, whereas secondary mutations in the activation loop were identified in half of these cases. This finding is consistent with a literature report in which cells harboring a primary *KIT* exon 9 mutation were exposed in vitro to sunitinib, and the resistant clones that grew out were found to harbor secondary *KIT* mutations located exclusively in the activation loop [23]. In contrast, secondary mutations in the ATP-binding pocket were indeed identified in the context of a primary *KIT* exon 11 mutation in the current study. Although PFS favored regorafenib (versus placebo) in patients in whom a mutation in the ATP-binding pocket was identified via BEAMing of fresh baseline ctDNA (Fig. 2B), this finding needs to be interpreted with particular caution on account of the relatively small number of these cases comprising this cohort (a correlation of individual secondary mutation with efficacy was not performed in the current analysis due to the low frequency of each individual mutation). SafeSEQ allows the identification of de novo mutations at a detection level (< 0.1% MAF [21]) that is not reached by hybrid capture NGS methods, which typically detect mutations at the 1% level [32]. The approach taken in this study therefore enabled the identification of a large variety of mutations, particularly in exon 17, which provides a sound understanding of the heterogeneity in *KIT* mutations in this late-line GIST setting. Consequently, this feature makes it particularly attractive to investigate potential treatment-induced changes in *KIT* mutations. Mutational changes during drug exposure to regorafenib were heterogeneous, with no specific pattern of resistance identified. Both increases and decreases in mutant allele fraction during treatment were observed that were independent of OS or tumor growth rate. The absence of a specific mutation pattern suggests that clonal selection is not a reason as to why these patients progressed on treatment.

Interestingly, this study also showed that a large number of patients were *KIT* wild type at baseline and remained so during treatment. Furthermore, in the 12 patients where the mutational status at baseline was wild type, the appearance of an activation loop mutation occurred in only one patient, and we therefore propose that the selection of a particular secondary mutation in exon 13/14 or exon 17/18 is not the standard resistance mechanism induced by regorafenib. This result therefore strongly suggests that an outgrowth of particular clones harboring a mutation conferring resistance to regorafenib is not the underlying reason for disease progression. However, it is conceivable that oncogenic alterations in the *KIT* ectodomain that were not part of this molecular analysis might play a role in driving tumor growth, analogous to what has been observed for the ErbB family of receptor tyrosine kinases [33].

It is important to bear in mind that this was an exploratory and retrospective study, and therefore hypothesis-generating in nature, and the potential impact of low-variant allele frequency mutations is yet to be established. In addition, although highly sensitive, the sequencing techniques used in this analysis have their limitations that have been acknowledged (e.g. in terms of discordance between BEAMing and SafeSEQ). Every effort has been made to mitigate these limitations, such as using more than one technique to analyze samples.

Recently, a variety of novel TKIs have entered clinical development for GIST. One of these is crenolanib, a novel inhibitor of type III receptor tyrosine kinases, which is currently in phase 3 development for *PDGFRA* D842V mutant GIST (NCT02847429) [34]. Another inhibitor, avapritinib (BLU-285), was developed as a selective inhibitor for mutant *KIT* and recently received FDA approval for the treatment of advanced GIST harboring a *PDGFRA* exon 18 mutation [11, 35]. A third inhibitor, ripretinib (DCC-2618), was designed to inhibit the full spectrum of mutant *KIT* and *PDGFRA* kinases in cancers [10]. Following encouraging results in a phase 1 trial [36], ripretinib is being evaluated versus sunitinib in a phase 3 trial in GIST following imatinib (NCT03673501). The ctDNA analysis technologies employed in this study may also be useful in the study of potential associations of mutational status with response in these ongoing studies. It remains to be seen if *KIT* mutation-specific inhibitors offer superiority over unselective inhibitors, such as regorafenib, or whether combinations of agents with complementary activity offers the best solution to combat resistance [12].

In summary, these data suggest that regorafenib may be able to secure long-term treatment benefit by avoiding manifestation of selective resistance mutations and support the positive outcome results of the GRID trial.

## Supplementary Information

Below is the link to the electronic supplementary material.

Supplementary file1 (DOCX 198 KB)

## Data Availability

Availability of the data underlying this publication will be determined later according to Bayer’s commitment to the EFPIA/PhRMA “Principles for responsible clinical trial data sharing”. This pertains to scope, time point and process of data access. As such, Bayer commits to sharing upon request from qualified scientific and medical researchers patient-level clinical trial data, study-level clinical trial data, and protocols from clinical trials in patients for medicines and indications approved in the United States (US) and European Union (EU) as necessary for conducting legitimate research. This applies to data on new medicines and indications that have been approved by the EU and US regulatory agencies on or after January 01, 2014. Interested researchers can use www.clinicalstudydatarequest.com to request access to anonymized patient-level data and supporting documents from clinical studies to conduct further research that can help advance medical science or improve patient care. Information on the Bayer criteria for listing studies and other relevant information is provided in the Study sponsors section of the portal. Data access will be granted to anonymized patient-level data, protocols and clinical study reports after approval by an independent scientific review panel. Bayer is not involved in the decisions made by the independent review panel. Bayer will take all necessary measures to ensure that patient privacy is safeguarded.
