# Supplementary material for: Broad spectrum of regorafenib activity on mutant KIT and absence of clonal selection in gastrointestinal stromal tumor (GIST): correlative analysis from the GRID trial
Source: Gastric Cancer. 2022 Jan 20;25(3):598–608. doi: 10.1007/s10120-021-01274-6 (PMC9013336; doi:10.1007/s10120-021-01274-6)
Supplement: Supplementary file 1 — Supplementary file1 (DOCX 198 KB) [file 10120_2021_1274_MOESM1_ESM.docx]

**Supplementary Material**

**Fig. S1. Mutations with known or likely oncogenic function in archival tumor tissue in patients enrolled in GRID as analyzed by NGS (n=43*)**


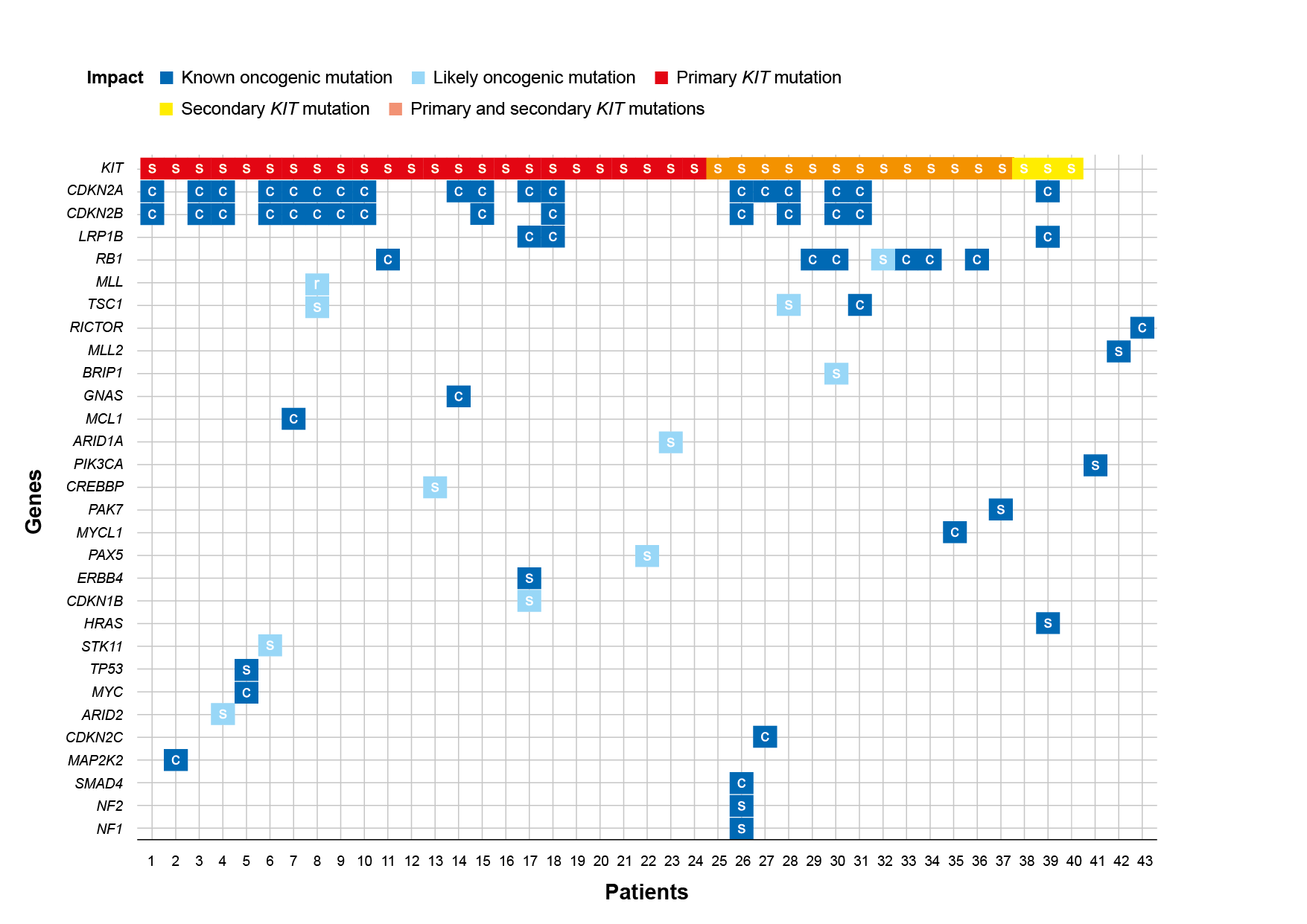

*Patients without any identified mutations (n=4) are not shown.
c, copy number alteration; r, rearrangement; s, short variants.

**Fig. S2. Kaplan–Meier plots of PFS in patients with the *KIT* exon 10 M541L variant (A) vs M541 (B) GIST as determined by SafeSEQ of plasma DNA**

**A)**

**
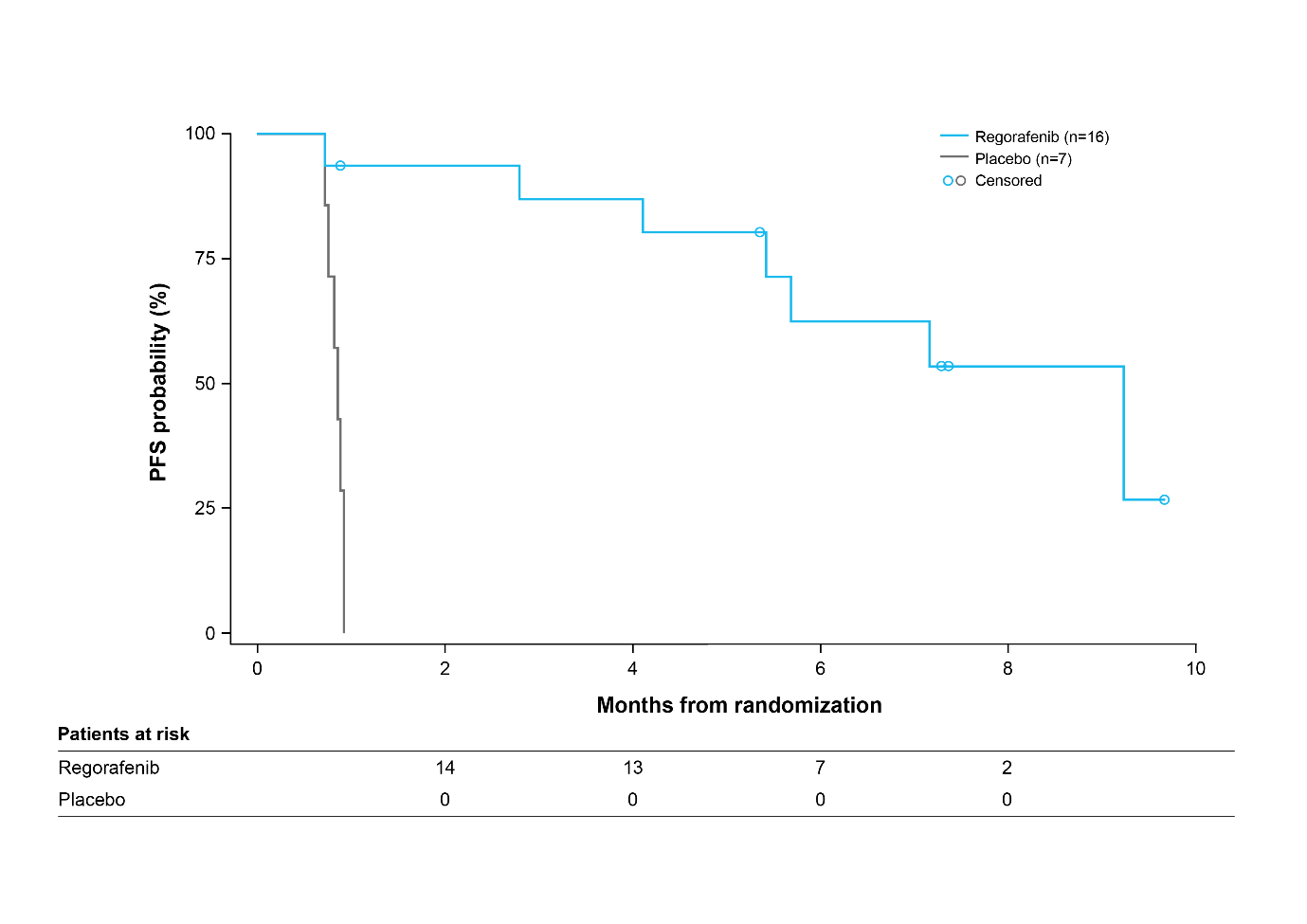
**

**B)**


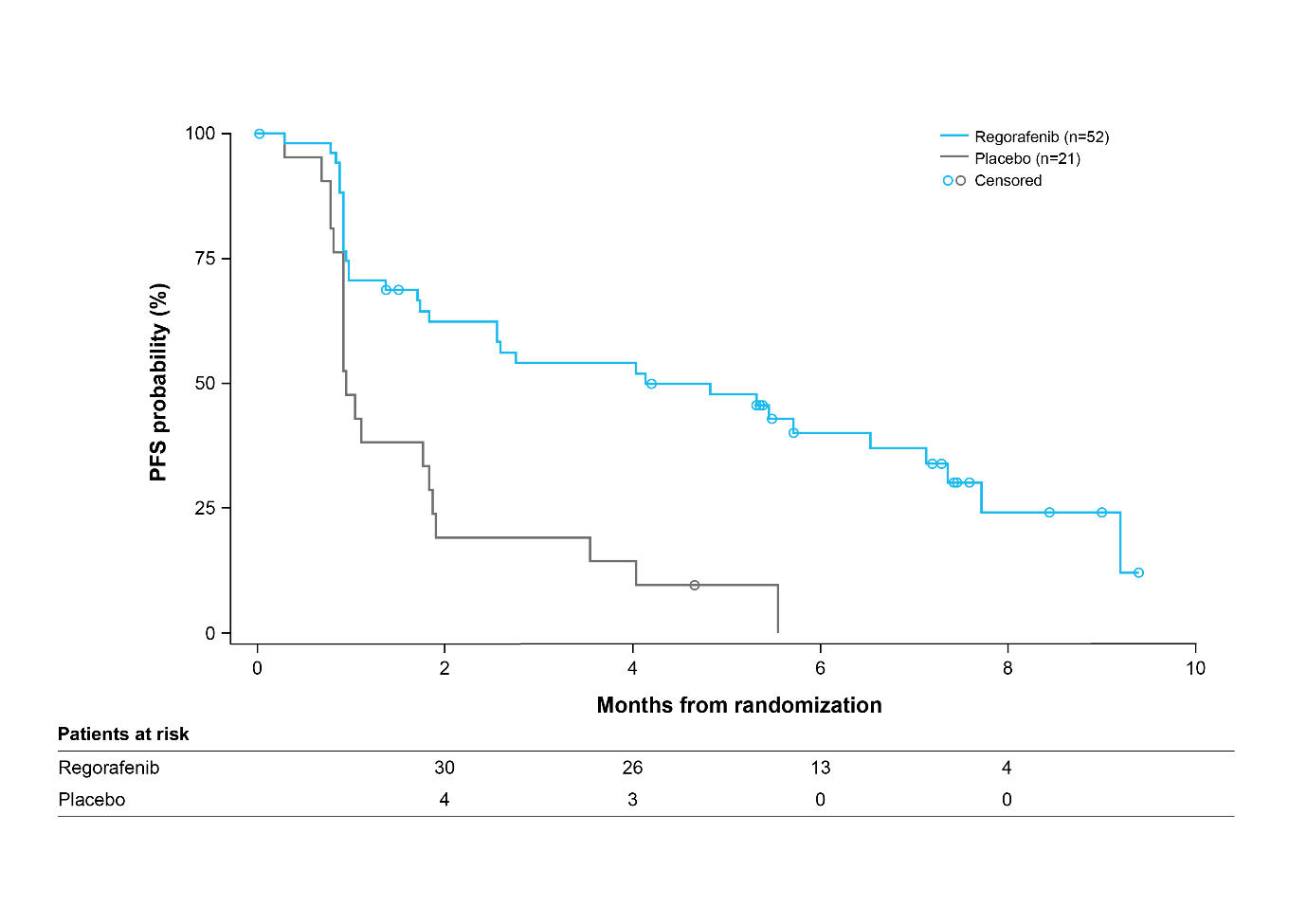


PFS, progression-free survival.

**Table S1. Mutations analyzed by BEAMing (plasma)**

| Gene | Exon | Nucleotide Change | Amino Acid Change |
| --- | --- | --- | --- |
| *KIT* | 9 | Insertion: GCCTAT at 1509 | Insertion: AY502-503 |
|  | 11 | Deletion: TGGAAG at 1669 | Deletion: W557-K558 |
|  | 11 | T1676C | V559A |
|  | 11 | T1676A | V559D |
|  | 11 | T1679A | V560D |
|  | 11 | T1727C | L576P |
|  | 13 | A1924G | K642E |
|  | 13 | T1961C | V654A |
|  | 14 | C2009T | Y670I |
|  | 14 | AC2008-2009GA | T670E |
|  | 17 | T2425G | C809G |
|  | 17 | G2446C | D816H |
|  | 17 | A2447T | D816V |
|  | 17 | A2447G | D816G |
|  | 17 | A2447C | D816A |
|  | 17 | C2448G | D816E |
|  | 17 | G2458T | D820Y |
|  | 17 | A2459G | D820G |
|  | 17 | A2459T | D820V |
|  | 17 | A2459C | D820A |
|  | 17 | A2460A | D820E |
|  | 17 | T2460G | D820E |
|  | 17 | A2464T | D822Y |
|  | 17 | A2464G | D822D |
|  | 17 | A2464C | D822H |
|  | 17 | T2466G | D822K |
|  | 17 | T2466A | D822K |
|  | 17 | T2467G | Y823D |
|  | 18 | G2485C | A829P |
| *PDGFRA* | 12 | T1682A | V561D |
|  | 15 | C2021T | T674I |
|  | 18 | A2525T | A842V |
|  | 18 | Deletion: 2524-2535 | Deletion: D842-H845 |
|  | 18 | Deletion: 2527-2538 | Deletion: I843-D846 |
| *BRAF* | 15 | T1799A | V600E |
| *KRAS** | 1 | G34A | G12S |
|  | 1 | G34C | G12R |
|  | 1 | G34T | G12C |
|  | 1 | G35A | G12D |
|  | 1 | G35C | G12A |
|  | 1 | G35T | G12V |
|  | 1 | G38A | G13D |

*Evaluated in plasma DNA from 2 subjects in whom a *KRAS* mutation was identified in tumor tissue.

**Table S2. Association of baseline plasma DNA levels with baseline tumor burden and efficacy endpoints (OS and PFS)**

| Quartile | N | DNA per mL of plasma* | Average baseline tumor burden† | *p*-value‡ | PFS HR (95% CI) | OS  HR (95% CI) |
| --- | --- | --- | --- | --- | --- | --- |
| 1 | 41 | 1,095 | 193 |  | 0.32 (0.12–0.88) | 1.13 (0.49–2.61) |
| 2 | 40 | 2,283 | 210 | NS | 0.40 (<0.01–0.36) | 0.53 (0.22–1.27) |
| 3 | 41 | 4,700 | 239 | NS | 0.21 (0.08–0.53) | 0.84 (0.41–1.71) |
| 4 | 40 | 24,996 | 275 | 0.042 | 0.38 (0.15–0.93) | 0.72 (0.33–1.55) |

*Average quantity of DNA (measured in genomic equivalents) per mL of baseline plasma obtained at enrollment into GRID.

†Sum of tumors existing at GRID enrollment as determined by a central laboratory.

‡A t-test comparing average tumor burden in the indicated quartile to that in the first quartile.

CI, confidence interval; HR, hazard ratio; NS, not significant; OS, overall survival; PFS, progression-free survival.

**Table S3. Identification of KIT alterations by exon in patient-matched samples by BEAMing only, SafeSEQ only, or both**

|  | **Exon 9** | **Exon 11** | **Exon 13** | **Exon 14** | **Exon 17** | **Exon 18** |
| --- | --- | --- | --- | --- | --- | --- |
| Total number of mutations detected | 14 | 39 | 15 | 7 | 76 | 15 |
| **Number of mutations detected by both BEAMing and SafeSEQ, n (%)** | **12 (86)** | **9 (23)** | **10 (67)** | **3 (43)** | **32 (42)** | **6 (40)** |
| Number of mutations detected by BEAMing **only,** n | 1 | 4 | 2 | 0 | 1 | 2 |
| Number of mutations detected by Safe SEQ **only,** n | 1 | 26 | 3 | 4 | 43 | 7 |

**Table S4. Sequence variation and genotype frequencies in *KIT* in patients with matched baseline and EoT plasma samples (n=41)**

| Patient | Baseline sequence variation | Frequency (%) | EoT sequence variation | Frequency (%) | Treatment |
| --- | --- | --- | --- | --- | --- |
| Duration of treatment >130 days (>4.3 months) | | | | | |
| 1 | p.W557_V559>C p.D820E  Not detected p.N822K  Not detected | 2.0  1.4  1.4 | p.W557_V559>C  p.D820E  p.Y823D  p.N822K  p.R830P | 16  0.21  13  0.21  0.31 | R |
| 2 | p.M541L**  p.D820Y  p.N822K | 42  1.5  0.12 | p.M541L**  p.D820Y  Not detected | 50  0.20 | P-R |
| 3 | p.M541L**  p.L576P  p.D820Y | 47  0.19  0.08 | p.M541L**  p.L576P  p.D820G  p.D820V | 48  0.48  0.10  0.16 | R |
| 4 | wildtype |  | p.829A>L_fs | 0.03 | P |
| 5 | wildtype |  | p.E554G  p.H650R | 0.01  0.01 | R |
| 6 | not detected  p.557delWK  not detected  not detected | 0.1 | p.V654A  p.557delWK  p.M536T  p.G424D | 1.21  1.38  0.02  0.02 | R |
| 7 | p.M541L  p.V559G | 45.3  0.1 | p.M541L  p.C673R  p.V559G  p.L644P  p.Y568H | 46.85  0.01  0.11  0.01  0.01 | R |
| 8* | wildtype | | wildtype | | R |
| 9* | wildtype | | wildtype | | R |
| 10 | wildtype | | p.D820Y  p.D820G | 0.60  0.30 | R |
| 11* | wildtype | | wildtype | | R |
| 12 | wildtype | | wildtype | | R |
| 13 | p.D820Y  p.R815I | 0.38  0.42 | Not detected  p.R815I | 0.37 | P-R |
| 14 | p.557delWK  p.D820G  p.Y823D  p.A829P | 23  19  1.3  0.20 | Not detected  p.D820G  p.Y823D  p.A829P | 0.39  0.14  0.13 | R |
| 15 | p.M541L**  p.557delWK  p.V654A  Not detected | 42  2.2  0.84 | p.M541L**  p.557delWK  p.V654A  p.C809G | 29  0.27  33  0.10 | R |
| 16* | wildtype | | wildtype | | R |
| 17 | p.V559G  Not detected | 1.4 | p.V559G  p.V654A | 0.33  0.30 | R |
| 18 | p.N822Y  p.N822K | 2.1  0.09 | p.N822Y  Not detected | 0.16 | P-R |
| 19* | wildtype | | wildtype | | R |
| Duration of treatment >90–≤130 days (>3–≤4.3 months) | | | | | |
| 20 | p.M541L**  Not detected  p.D820Y  p.D820E  p.N822K  p.Y823D | 44  0.52  0.55  0.55  0.51 | p.M541L**  p.815delRD  Not detected Not detected Not detected  p.Y823D | 48  0.10  0.07 | P-R |
| 21 | Not detected  Not detected  p.V559D  p.V654A  p.D677N | 2.6  1.9  0.16 | p.I571R  p.D579V  p.V559D  p.V654A  p.D677N | 4.3  4.3  15  12  1.4 | R |
| 22 | Not detected p.D820G  p.A829P | 0.81  0.43 | p.N822K  p.D820G  p.A829P | 0.08  0.20  0.59 | R-R |
| 23 | p.D816E  p.Y823D  p.N822K  not detected | 3.2  0.9  0.4 | p.D816E  p.Y823D  p.N822K  p.A829P | 4.21  3.11  0.10  5.55 | R |
| Duration of treatment ≤90 days (≤3 months) | | | | | |
| 24 | p.557WKV>F  p.A829P | 6.1  0.19 | Not detected | | R |
| 25* | wild-type | | wild-type | | R |
| 26 | p.V654A | 1.0 | p.V654A | 3.1 | R |
| 27 | p.501insAY  p.D820G  p.D820H  Not detected | 8.8  1.5  8.4 | p.501insAY  p.D820G  p.D820H  p.D816E | 23  0.17  26  0.07 | R |
| 28 | wildtype |  | p.V654A  p.C673R  p.559delV | 0.07  0.01  0.15 | R-R |
| 29 | p.N822Y  p.A829P | 0.6  0.9 | p.V654A  p.N822K  p.D820G  p.A829P  p.A795T | 0.28  0.02  0.03  1.63  0.01 | R |
| 30 | p.501insAY  p.M541L  p.N680K  not detected  not detected  p.F681I  p.C809G  not detected  p.D816G  p.D820N  not detected  p.D820G  p.N822Y  p.N822I  p.N822K | 13.3  53.1  0.2  0.2  0.2  0.3  0.4  0.1  0.4  1.6  3.2 | p.501insAY  not detected  not detected  p.K642T  p.648insN  p.F681L  p.C809G  p.C809S  not detected  not detected  p.D820Y  p.D820G  p.N822Y  p.N822T  p.N822K | 8.60  0.03  0.07  0.25  0.43  0.02  0.11  0.07  1.51  0.46  0.17 | R |
| 31 | not detected  not detected  not detected  p.558K>NQ  not detected  not detected | 9.8 | p.L644P  p.D820Y  p.Y503F  p.558K>NQ  p.A829P | 0.01  0.01  0.09  13.06  0.26 | R |
| 32 | p.501insAY  p.F681L | 0.4  0.4 | p.501insAY  not detected | 0.08 | R-R |
| 33 | p.D820Y  not detected  not detected  not detected | 3.3 | p.D820Y  p.D820A  p.D820G  p.A795T | 10.01  0.04  0.11  0.01 | R-R |
| 34 | p.557delW  p.W557*  p.V654A | 19.4  17.6  0.2 | p.557delW p.W557*  not detected | 3.19  3.18 | R |
| 35 | p.M541L | 57.6 | p.M541L | 61.70 | R |
| 36 | p.V654A  not detected  not detected | 1.2 | p.V654A  p.D419G  p.G534S | 11.92  0.01  0.01 | R-R |
| 37 | not detected  p.A829P | 26.3 | p.M651V  p.A829P | 0.01  0.12 | R-R |
| 38 | not detected  not detected  not detected  p.501insAY  not detected  p.A829P | 0.4  0.5 | p.D820Y  p.C673R  p.N822K  p.501insAY  p.V569F  not detected | 0.35  0.02  0.15  0.46  0.01 | R-R |
| 39 | p.K509I  p.V654A  p.N822K  not detected | 0.1  2.6  0.2 | p.V654A  p.Y823D  p.D820Y  p.N822K  p.557delWKV | 0.04  1.79  0.03  14.66  18.92 | P-R |
| 40 | wildtype |  | p.N567D  p.C809R | 0.01  0.02 | P-R |
| Duration of treatment unknown | | | | | |
| 41 | p.501insAY  p.C809G | 2.4  3.1 | p.501insAY  Not detected | 0.08 | R |

*Patients with wild-type *KIT* at baseline and EoT.
**M541L is a germline polymorphism, not a tumor-associated mutation.
EoT, end of treatment; P-R, crossed over to open-label regorafenib after progression; R, double-blind regorafenib; R-R, continued open-label regorafenib after progression.
